# Supplementary material for: Chromothripsis during telomere crisis is independent of NHEJ, and consistent with a replicative origin
Source: Genome Res. 2019 May;29(5):737–49. doi: 10.1101/gr.240705.118 (PMC6499312; doi:10.1101/gr.240705.118)
Supplement: Supplemental Material [file supp_gr.240705.118_Supplemental_file_1.zip › contigs/annotated_contigs/DB68/contig.2.DB68_length_473_mean_cov_9.96405919662.docx]

**DB68_length_473_mean_cov_9.96405919662**

```
CTAATAATACAAAAAATTAGCTGGGCATGGTG|ATGCACT|CCTGTAATCCCAGCTACTCGGGAGGCTGAGGCAGGAGAATCGCTTGAA
 >chr20:31259952-31259984 + E=1e-07     >chr3:183190496-183190931 - E=1e-209
CCCAGGAGGCAGAGGTTGCGGTGAGCCGAGATCGCGCCACTGCACTCCAGTCTGGGCAACAAGAGCGAAACTCCATTAAAAAAAATGAG

AACAAGGGCTAGCAAAACGCAGGAGGTAGCAAAACACAAATGATTCCAAACCTCAGACAGTGAAGCAAATGCTCTTCCTTCTTGGATTG

CTTCTCTTGACCCCACCCACCTGTTCCCCTGCCCTTTCCTTCTGATGGGAACTTGGGATGATCCGTGCTGCATGGGGGAGGAAGGGCAG

GTGAGGAGGGACGGGACCTGGGCTGACCAGCTAGCTGTTCTGAGCTGCCATGGGGTCTGTCTGGAATTTATAGACATTAGGTGTTAACC

TGGTATCTCTGTGCCTCAGTTTCTTCTTCT
```
